# Calcium Phosphate Nanoparticles as Carriers of Low and High Molecular Weight Compounds

**DOI:** 10.3390/ijms252312887

**Published:** 2024-11-29

**Authors:** Ekaterina Popova, Victoria Tikhomirova, Assel Akhmetova, Irina Ilina, Natalia Kalinina, Michael Taliansky, Olga Kost

**Affiliations:** 1 Shemyakin-Ovchinnikov Institute of Bioorganic Chemistry, Russian Academy of Sciences, 117997 Moscow, Russia; popova.ekaterina1995@gmail.com (E.P.); vetikhomirova@gmail.com (V.T.); akhmetovaai@my.msu.ru (A.A.); ilina@ibch.ru (I.I.); kalinina@belozersky.msu.ru (N.K.); michael.taliansky@hutton.ac.uk (M.T.); 2 Chemistry Faculty, Lomonosov Moscow State University, 119991 Moscow, Russia; 3 Physical Faculty, Lomonosov Moscow State University, 119991 Moscow, Russia; 4 Belozersky Institute of Physico-Chemical Biology, Lomonosov Moscow State University, 119234 Moscow, Russia; 5 The James Hutton Institute, Invergowrie, Dundee DD2 5DA, UK

**Keywords:** calcium phosphate nanoparticles, enalaprilat, superoxide dismutase, DNA, RNA

## Abstract

Nanoparticles could improve the bioavailability of active agents of various natures to human, animal, and plant tissues. In this work, we compared two methods on the synthesis of calcium phosphate nanoparticles (CaPs), differed by the synthesis temperature, pH, and concentration of the stabilizing agent, and explored the possibilities of incorporation of a low-molecular-weight peptide analogue enalaprilat, the enzyme superoxide dismutase 1 (SOD1), as well as DNA and dsRNA into these particles, by coprecipitation and sorption. CaPs obtained with and without cooling demonstrated the highest inclusion efficiency for enalaprilat upon coprecipitation: 250 ± 10 μg/mg of CaPs and 340 ± 30 μg/mg of CaPs, respectively. Enalaprilat sorption on the preliminarily formed CaPs was much less effective. SOD1 was only able to coprecipitate with CaPs upon cooling, with SOD1 loading 6.6 ± 2 μg/mg of CaPs. For the incorporation of DNA, the superiority of the sorption method was demonstrated, allowing loading of up to 88 μg/mg of CaPs. The ability of CaPs to incorporate dsRNa by sorption was also demonstrated by electrophoresis and atomic force microscopy. These results could have important implications for the development of the roots for incorporating substances of different natures into CaPs for agricultural and medical applications.

## 1. Introduction

Calcium phosphate is the major mineral constituent of bones and teeth in mammals, giving these organs hardness. Being a component of the human body, calcium phosphate is biocompatible and biodegradable, which makes it a promising material for medical use. Among the different modifications of calcium phosphate, calcium-deficient hydroxyapatite possesses the most similarity to the mineral part of bone. It is interesting that biologically formed under mild conditions calcium phosphate (biological apatite) is always nanometric and nanocrystalline. The crystal dimensions of biological apatite in the calcified tissues always possess a range of a few to hundreds of nanometers with the smallest building blocks on the nanometer size scale [1]. Thus, artificially created nanometric calcium phosphate forms (CaPs) are able to mimic the constituent components of the calcified tissues. This is why bone tissue engineering is the field in which calcium phosphates are mainly studied to get the ideal implants to regenerate bone tissue while it is resorbed. Applications of CaPs include the coating of dental implants [2], the treatment of spinal injuries [3], orthopedics [4], tissue engineering [5], etc. However, calcium phosphate systems, especially CaPs, are prospective inorganic materials for a more wide range of applications. They can be used as efficient carriers for various kinds of biomolecules, such as oligonucleotide and plasmid DNA, miRNA, proteins, peptides, or drugs, for the delivery to different cells and tissues where their action is required [6,7,8,9,10,11,12]. It is important that CaPs are readily soluble at low pH inside endolysosomes or phagosomes, that is, after cellular uptake, but stable at neutral pH, for example, in the blood. CaPs inside the body do not pose a risk as they are typically resorbed and dissolved by osteoclasts and macrophages [13].

In addition, CaPs themselves contain macronutrients for plants, so CaPs are often used in agronomy as fertilizer [14]. Moreover, CaPs can be dopped by additional plant-friendly compounds, i.e., urea [15,16], zinc ions [17], and methyl jasmonate [16], etc.

The use of CaPs as potential carriers of peptides, DNA, and RNA is of special interest for their application in the crop industry. The problem of agricultural losses due to plant diseases and pests is currently relevant: losses range from 17 to 30% depending on the type of crop [18]. Current approaches to control plant pathogens and diseases rely on the use of chemicals, insecticides, and fungicides, or the development of genetic disease resistance by breeding transgenic varieties. While fungicides and insecticides can be toxic to other organisms in the environment, the use of exogenous double-stranded RNA (dsRNA) to induce RNA interference can be a new tool for plant protection via a post-transcriptional gene silencing mechanism triggered upon recognition of dsRNA to suppress homologous messenger RNA [19].

Different methods for the synthesis of CaPs with different size and composition were developed [1,20,21,22]. These methods allow the synthesis of CaPs of nanosizes in more or less agglomerated form. In general, CaPs are synthesized by various methods like precipitation from water solutions [23,24,25,26,27,28,29,30,31], flame spray pyrolysis and from biogenic resources [21], in the system of reversed micelles [32], etc. CaPs usually form suspensions in water but tend to precipitate easily from concentrated solutions. The precipitation from water is easy, cost-efficient, and environmentally friendly, as no organic solvent is required. The synthesis of CaPs is also carried out in the presence of polymers, as well CaPs can be additionally covered by lipids, simple sugars or chitosan [27,31,33]. In general, the loading of CaPs by bioactive agents could be achieved either by coprecipitation, that is during CaPs synthesis, or by sorption. One of the simplest methods of the CaPs synthesis is mixing of the salts of calcium and phosphate in water solution in the presence of stabilizing agent, sodium citrate, under constant ultrasonic treatment with or without further maturation of the obtained CaPs [31,34]. Also, this method is extremely simple and cheap, it required ultrasonic treatment to obtain stable nanosized CaPs. It could be a problem for the inclusion of thermolabile compounds into CaPs during the synthesis. For these purposes, cooling of the reaction mixture might solve the problem. However, the temperature is one of the factors influencing CaPs characteristics [1,20], which, in turn, would require the changing some other synthesis conditions. Besides, it would be interesting to explore the possibility of two types of CaPs obtained under different conditions to incorporate the compounds of different nature. For this purpose, we used two approaches: (1) direct inclusion of compounds during CaPs synthesis, i.e. by coprecipitation; (2) the adsorption of these compounds on preliminarily synthesized CaPs.

Thus, the aim of this work was a comparison of two types of CaPs obtained at different conditions forming stable suspensions and allowing both stable and labile compounds to bind, namely, low molecular weight tripeptide analogue (enalaprilat), an enzyme superoxide dismutase 1, DNA, and double-stranded RNA, by coprecipitation and by sorption.

## 2. Results and Discussion

### 2.1. Synthesis and Characteristics of Calcium Phosphate Nanoparticles

Since CaPs could serve as carriers of a wide range of substances, both thermally stable and labile, we compared two different methods of CaP synthesis, differed by the temperature and pH of the synthesis medium, as well as the concentration of a stabilizing agent, sodium citrate. We always synthesized the particles with ultrasound exposure so that they did not have a tendency to form a precipitate, but instead formed a stable suspension. This is extremely important for their further use in both medicine and agriculture, so that they can be applied as suspensions by instillation, dripping, or spraying.

It is known that sodium citrate can serve as a stabilizing agent for CaPs [34], as it forms a charged layer around the nanoparticles and prevents their coagulation. So, we varied sodium citrate concentration to find the proper conditions of CaP formation. These experiments were carried out at fixed concentrations of stock solutions of potassium phosphate and calcium chloride and at fixed power and time of ultrasonic treatment without any precautions, that is, without any cooling of the reaction mixture under ultrasonic treatment. We have shown that the presence of sodium citrate is absolutely necessary for proper CaP formation, as in the absence of citrate or at low citrate concentrations, only large particles with substantial precipitation occurred, likely due to aggregates formation (Table 1). The increase in the sodium citrate concentration up to 7.8 mM allowed us to obtain the nanosized particles with a mean hydrodynamic diameter of about 210 nm (Table 1), whereas a further increase to 15.6 mM sodium citrate led to a further significant decrease in the mean hydrodynamic diameter (about 80 nm) of the obtained CaPs measured by dynamic light scattering (DLS). Higher concentrations of sodium citrate did not further decrease CaP size, while ζ-potential of CaPs was virtually the same at any sodium citrate concentration (Table 1).

Then, we synthesized CaPs at 15.6 mM sodium citrate but at two different temperature conditions: (a) without cooling as above; in these conditions the temperature of the system rose from room temperature to 60–70 °C at the end of a constant ultrasonication; (b) with ice cooling of a reaction mixture during all ultrasonication time, which resulted in a temperature rise of 20–23 °C towards the end of the treatment. Additionally, we varied the pH values of the stock solution of potassium chloride in both cases. We showed that the synthesis without cooling led to the formation of a stable suspension of CaPs. The mean hydrodynamic diameter of CaPs measured by DLS was lower at higher pH values, and the polydispersity index of the system indicated more uniform CaP formation (Table 2). However, CaPs synthesized at low temperature were characterized by low stability, as some precipitation was observed at all pH values of the reaction medium (Table 2).

We showed that increasing the concentration of the stabilizing agent citrate allowed us to increase the stability of the system when obtaining CaP particles without cooling (Table 1). Therefore, we hypothesized that increasing the citrate content would also affect the stability of CaPs synthesized upon cooling. Indeed, a two- and three-fold increase in the initial concentration of sodium citrate helped to obtain the stable suspension of CaPs with appropriate sizes without any precipitate (Table 3). Note that pH values were checked at each stage of the synthesis. The different concentrations of sodium citrate used in this study did not influence the final pH value of the system. It is interesting, however, that at higher pH of the stock solution of potassium phosphate, the mean hydrodynamic diameter of the CaPs was higher, in contrast to what was observed when CaPs were obtained without cooling (see Table 2).

**Figure 1:**
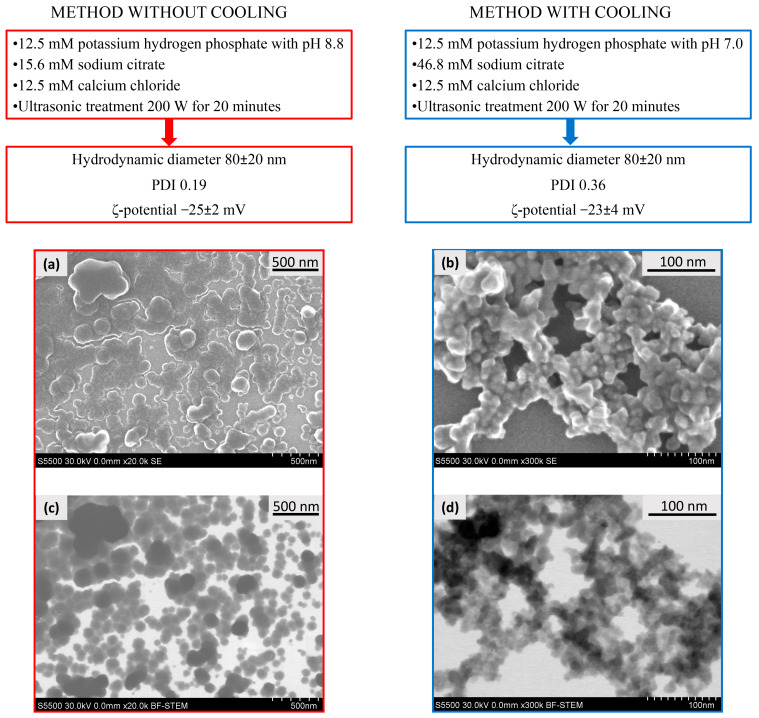
The conditions of CaP synthesis characteristics and microphotographs of CaPs formed at different conditions. SEM (**a**,**b**) and STEM (**c**,**d**) images of CaPs obtained without cooling (**left**), and obtained with cooling (**right**).

As a result, for further experiments, we selected two methods for the synthesis of CaPs (Figure 1) bearing in mind that a method without cooling could be further used for the inclusion of thermostable active agents in the coprecipitation stage, while a method with cooling could be useful for the inclusion of less stable biological molecules, i.e., nucleic acids and proteins. It should be especially noted that we use the term “coprecipitation” for the process where a substance can be included into CaPs during particles formation, i.e., in situ. Despite the fact that these systems did not form real precipitates in the chosen conditions, they formed stable suspensions. Additionally, we tried both types of CaPs as putative carriers of the substances which could not be included into the nanoparticles by coprecipitation but could be included by sorption.

It should be noted that CaPs obtained by the chosen methods were characterized by similar values of a mean hydrodynamic diameter and ζ-potential; but, the polydispersity index of the particles obtained with cooling was two times higher, that is, CaPs obtained with cooling were characterized by broader distribution in size (Figure 1). Additionally, CaP suspensions obtained under different conditions had different CaP content, determined by weight after dialysis: CaP concentration obtained without cooling was about 1 mg/mL, CaP concentration obtained with cooling was two-fold less: approximately 0.5 mg/mL. Apparently, this is due to the solubility of calcium phosphate, as the higher the pH of the system, the lower the solubility of calcium phosphate, and, accordingly, the more effective the precipitation of the salt at higher pH (method without cooling).

Both types of CaPs were characterized by scanning electron microscopy (SEM) (Figure 1a,b) and scanning transmission electron microscopy (STEM) (Figure 1c,d) methods. CaPs obtained without cooling were represented by a rounded shape with a size range of 20–150 nm (Figure 1a,c). In addition, the formation of CaP aggregates was observed upon drying on a carbon matrix. The average hydrodynamic diameter measured with dynamic light scattering (DLS) was consistent with the particle sizes in SEM and STEM images. STEM and SEM images of CaPs with cooling showed agglomerates consisting of nanocrystallites of 10–20 nm in size (Figure 1b,d). Apparently, the smaller size of separate particles with lower synthesis temperature could be observed due to the slower growth of the particles than at a high temperature. Despite the differences in morphology, the hydrodynamic diameters obtained by DLS were similar and corresponded to 80 nm for both particle types. Since the PDI value was twice as high for CaPs with cooling, it can be assumed that they exist in suspension as agglomerates of different sizes consisting of nanocrystallites. It is worth noting that we could observe a bright halo around individual CaPs obtained both with and without cooling, likely formed by the stabilizing agent, sodium citrate.

**Figure 2:**
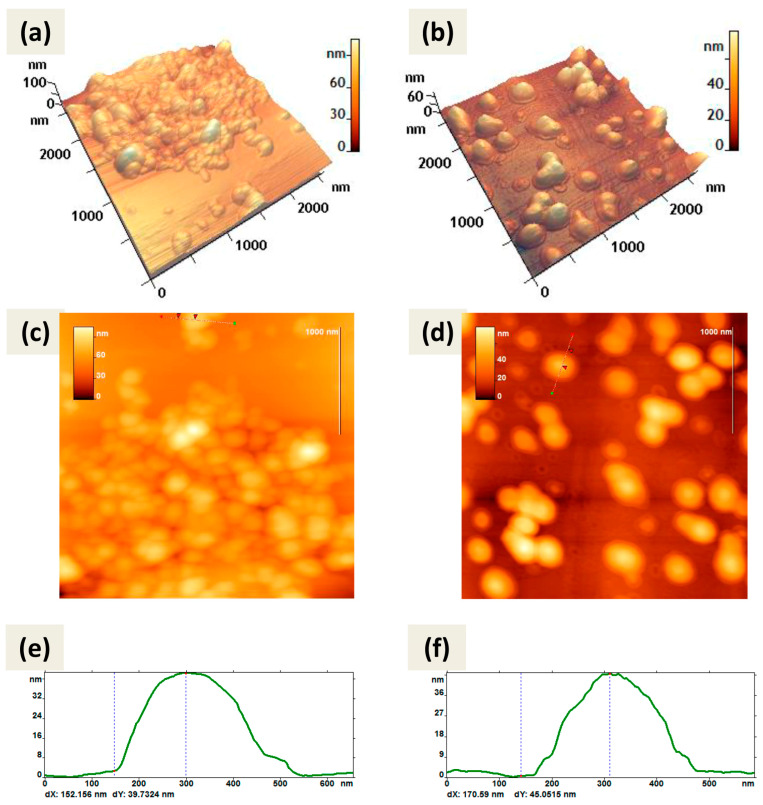
AFM images of CaPs, obtained without cooling on graphite (**left** column); CaPs, obtained with cooling on mica substrate (**right** column): (**a**,**b**) 3D image; (**c**,**d**) 2D image; (**e**,**f**) cross-section of the individual particle on 2D image. The red arrows (**c,d**) correspond to the dotted lines on the cross-section (**e**,**f**). The particle height according to the cross-section (**e**) is 39 nm; according to the cross-section (**f**) is 45 nm.

Atomic force microscopy (AFM) also showed predominantly spherical shapes of CaPs obtained by both methods (Figure 2). Both types of particles were not prone to destruction when exposed to a cantilever and were quite elastic and did not aggregate; when drying on graphite or mica they tend to form a monolayer.

Note that the method of CaP synthesis with cooling included increased amounts of the stabilizing agent, sodium citrate. So, we can attribute the observed in Figure 2b,d border to the citrate shell on the surface of CaPs. Some CaPs obtained without cooling at lower concentration of sodium citrate also showed such borders (Figure 2a) but not so obvious.

Using AFM, a detailed morphometric analysis of CaPs was carried out; for each sample of particles the parameter values were determined using FemtoScan Online software version 2.4.26. Table 4 shows the average values of CaP parameters obtained during the AFM study.

As can be seen from Figure 2 and Table 4, CaPs obtained with cooling had larger values of perimeter, area, and surface roughness. Based on the form factor values, both types of particles had an approximately similar shape; not ideally spherical, but rather oval. This may partly be a consequence of the peculiarities of adsorption on the surface. When comparing the value of the diameter and the average height, CaPs obtained with cooling tended to spread out more over the surface of the substrate.

**Figure 3:**
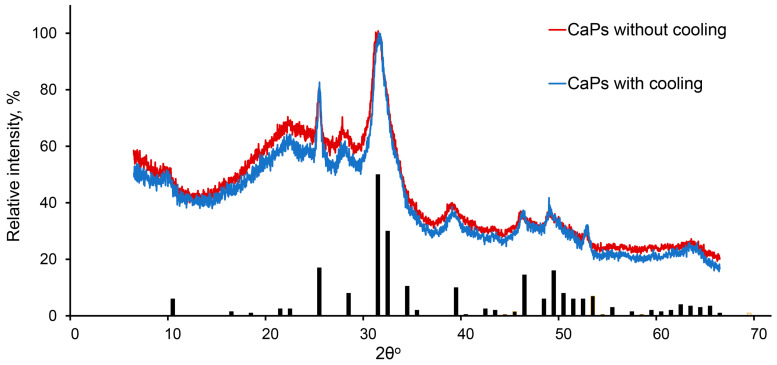
X-ray diffraction pattern of CaPs obtained without cooling (red) and with cooling (blue). The bars depict the characteristic spectrum of hexagonal hydroxyapatite.

It was shown previously that at higher pH values and higher temperature, hydroxyapatite is formed [35], while in a more acidic environment and lower temperature amorphous, calcium phosphate tends to be formed [36]. In our conditions, the phase composition of CaP samples obtained by two chosen methods turned out to be the same. They consisted of both hexagonal hydroxyapatite (Ca_10_(PO_4_)_6_(OH)_2_) and amorphous calcium phosphate (Ca_x_(PO_4_)_y_·zH_2_O), which could be determined by the broadening of the peaks corresponding to the crystalline hydroxyapatite phase (Figure 3).

Thus, we applied two methods of CaP synthesis—the first method was without cooling during ultrasonication, with a higher pH value of the stock solution of potassium phosphate and lower concentrations of sodium citrate; the second method was with ice cooling during ultrasonic treatment, with a lower pH value of the stock solution of potassium phosphate and higher concentrations of sodium citrate. Both methods led to a formation of nanoparticles which were characterized by similar phase composition, and similar mean hydrodynamic diameter and ζ-potential. Nevertheless, CaPs obtained at lower temperatures were smaller and more prone to aggregation.

Both types of CaPs, however, formed stable water suspensions which make them prospective for their use in different areas, including medicine and agriculture. So, CaPs appeared to be quite stable as suspensions in water and did not change the size and ζ-potential during storage for a month at 4 °C. The stability of CaPs in the physiological solution (0.15 M NaCl, pH 7.4) was lower, and the particles did not change their characteristics for two weeks; after that, aggregates began to form, although they were still soluble. Stability increased in the presence of albumin in the physiological solution, i.e., in a medium imitating tear fluid. Slow degradation of calcium phosphate in most human body fluids and cells is not a drawback but rather a merit, as this property does not allow the particles to accumulate somewhere in the organism with unexpected side-effects, while the stability of CaP suspensions in water provide a possibility of the use of such suspensions in the crop industry.

### 2.2. Inclusion of Different Substances into CaPs

#### 2.2.1. Low Molecular Weight Substance Inclusion

To test the capacity of CaPs obtained by the two methods, we incorporated a model low-molecular-weight active substance, the angiotensin-converting enzyme (ACE) inhibitor enalaprilat (MW 348.4 Da), at the stage of CaP formation (referred to as coprecipitation), and by the sorption on the preliminarily obtained CaPs (Table 5). We estimated the amount of enalaprilat bound with CaPs by a difference between the initial amount of enalaprilat and the amount found in filtrate after ultracentrifugation. As these values corresponded to the amounts of enalaprilat eluted from CaPs, we considered this approach as correct.

For the estimation of a weight load of enalaprilat, the corresponding amount of empty CaPs was washed with water, lyophilized, and weighed.

It turned out that the percentages of the inclusion of enalaprilat into CaPs obtained with and without cooling were different: 27 ± 4% and 40 ± 10%, respectively. Note that the contents of CaPs obtained with equal stock salt solutions, but using two methods described above, differed twice, with the content of CaPs with cooling being about two-fold lower. So, for the proper comparison of the capacities of two types of CaPs, we used equal ratios of enalaprilat/CaPs. The weight load of enalaprilat inclusion into CaPs without cooling was also higher (Table 5). The difference in this enalaprilat capacity is likely due to the size and morphology of CaPs obtained in different conditions (Figure 1), as the volume of individual particles obtained without any temperature precautions was larger than that of CaPs obtained with cooling.

The inclusion of enalaprilat into two types of CaPs provided a slight increase in hydrodynamic diameter to 110 ± 20 nm and a decrease in ζ-potential to −29 ± 2 mV. Enalaprilat-loaded particles remained completely stable (their hydrodynamic diameter and ζ-potential did not change) for two weeks, then they began to aggregate and increased their size to 200 nm after two months of storage. Meanwhile, their ζ-potential in absolute value decreased to −8 mV.

Unexpectedly, we did not detect any enalaprilat sorption on the preliminarily formed CaPs obtained with cooling, while the sorption on CaPs without cooling was rather high, although the percentage of the binding was much less (about 5-fold) than in the case of coprecipitation (Table 5) and was less than 10% of the initial amount.

Thus, a low-molecular-weight enalaprilat is likely able to be included in the structure of CaPs as a result of coprecipitation by interacting with Ca^2+^, so the efficiency of its inclusion in CaPs in this way was quite high. However, the properties of CaP surfaces seem to be unfavorable for enalaprilat binding, which can be explained by a negative charge of enalaprilat and negative ζ-potential of CaPs themselves, besides the fact that the CaPs obtained with cooling required a larger amount of the stabilization agent, sodium citrate.

#### 2.2.2. Protein Inclusion

To test the protein capacity of CaPs, we chose the enzyme, recombinant superoxide dismutase 1 (SOD1, MW 32.5 kDa), the inclusion of which could be followed both by protein and catalytic activity and tried to include this enzyme into CaPs obtained by the two methods: both by coprecipitation with CaPs and by sorption on the preliminarily obtained particles. As we expected, SOD1 failed to coprecipitate with CaPs without cooling: we observed intense foaming, and the enzyme irreversibly lost its catalytic activity due to the increase in temperature during ultrasonic treatment. However, SOD1 can be included into CaPs by coprecipitation with cooling, likely due to electrostatic coordination with calcium ions, although with a rather low yield of 11 ± 3%. The weight load of SOD1 into CaPs was equal to 6.6 ± 2 µg/mg particles. The loading of SOD1 into CaPs resulted in a slight increase in their hydrodynamic diameter from 80 nm to 110 ± 25 nm, and the ζ-potential remained unchanged.

The sorption of SOD1 on CaPs gave no result, likely because of the negative charge of SOD1 (pI about 4.8) under sorption conditions, as it was in the case of enalaprilat (see above). Thus, negative-charged enzyme SOD1 could only be included into CaPs by the coprecipitation technique.

SOD1-loaded CaPs remained stable (their hydrodynamic diameter and ζ-potential did not change) for at least two weeks, after which they started to aggregate, but they still retained suspension without precipitation. Loading SOD1 into CaPs allowed the enzyme to significantly prolong its activity during storage. SOD1 within CaPs reduced its activity to 95 ± 5% in a week, whereas SOD1 in an aqueous solution completely lost its activity by this time. Storing SOD1-loaded CaPs for two months reduced its enzymatic activity to 60 ± 10%.

#### 2.2.3. DNA Inclusion

We showed that in the presence of DNA (MW 270–500 kDa from salmon milt) CaPs did not form at all; that is, likely large DNA molecules were just a formation of obstacle nanoparticles. So, DNA inclusion into CaPs by coprecipitation is highly unlikely.

However, we found that DNA was able to bind effectively to CaPs by sorption, especially in the presence of additional metal ions. The interaction of nucleic acids and CaPs is mediated by the electrostatic attractions between Ca^2+^ and the phosphate groups of DNA or RNA [7,37,38,39], while additional ions can act as bridges between the nucleic acid and the surface of the carrier bearing a negative charge [40,41]. So, we chose a pair of divalent ions, Ca^2+^ and Mg^2+^, which were previously shown to enhance an adsorption of DNA to hydroxylapatite nanorods and a clay montmorillonite.

**Figure 4:**
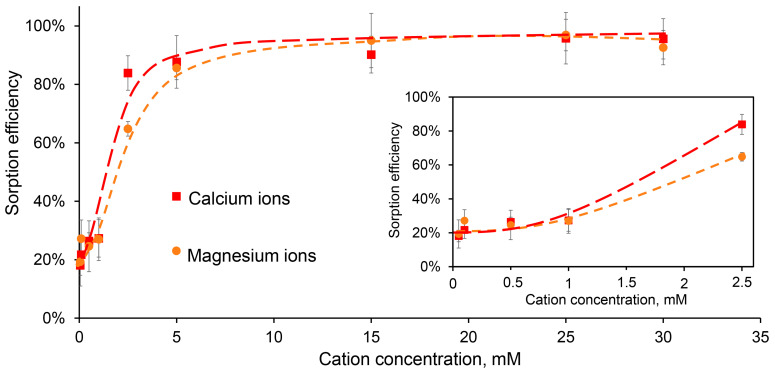
The efficiency of DNA sorption (50 μg/mL) on CaPs (0.93 mg/mL) in the presence of different concentrations of Ca^2+^ (red) and Mg^2+^ (orange). The sorption efficiency within the initial ion concentration range of 0.05–2.5 mM is inside the frame.

Thus, we studied the sorption of DNA on CaPs obtained without cooling in the presence of additional concentrations of Ca^2+^ and Mg^2+^. It appeared that at low-ions concentrations, less than 5 mM, the efficiency of DNA sorption was also low (Figure 4).

Overall, the difference between the effects of Ca^2+^ and Mg^2+^ on DNA sorption on CaPs was statistically insignificant, with the exception of the concentrations around 2.5 mM, where Ca^2+^ was more effective (Figure 4). It should be noted that in these experiments we used a fixed DNA concentration of 50 μg/mL, and increasing it to 100 μg/mL reduced the relative efficiency of sorption (Table 6). For further experiments, a concentration of 5 mM was chosen as a minimal additional divalent ion concentration when almost complete DNA binding to CaPs was observed.

**Figure 5:**
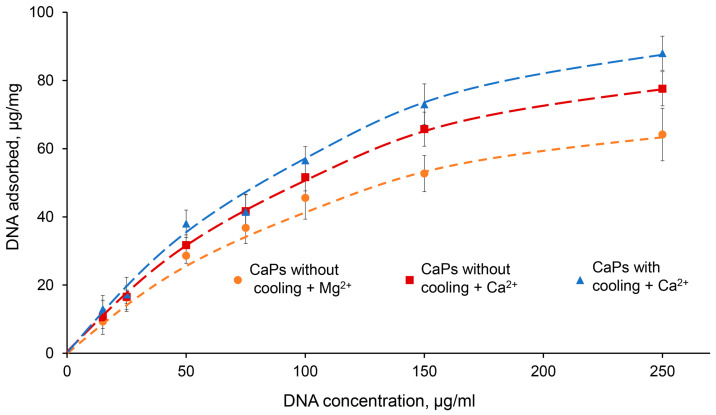
Sorption isotherms of DNA (15–250 μg/mL) on 1 mg CaPs in the presence of 5 mM Ca^2+^ and Mg^2+^. (Dependence of sorbed DNA mass on the initial DNA concentration per 1 mg of CaP particles). Orange—CaPs without cooling in the presence of Mg^2+^; red—CaPs without cooling in the presence of Ca^2+^; blue—CaPs with cooling in the presence of Ca^2+^.

In order to characterize DNA sorption on CaPs more completely, we studied the isotherm of DNA sorption on CaPs obtained with and without cooling under optimal conditions, that is in the presence of 5 mM Ca^2+^ or Mg^2+^ (Figure 5). Note that during the sorption of DNA on CaPs obtained with cooling in the presence of Mg^2+^, it was not possible to precipitate the suspension properly by centrifugation and, therefore, we could not determine the amount of unbound DNA, and the sorption isotherm could not be obtained for this case.

As can be seen from the graph, at low DNA concentrations (up to 50 μg/mL) there was practically no difference between the curves obtained for CaPs with and without cooling, as well as for different ions; that is, the presence of a higher concentration of citrate required for the synthesis of CaPs with cooling was not important for DNA sorption. However, at higher DNA concentrations, 150 and 250 μg/mL, where the amount of sorbed DNA on the particles was also significantly higher, Ca^2+^ demonstrated higher efficiency compared to Mg^2+^. This effect of its action was more pronounced in the case of CaPs obtained with cooling (Figure 5). The adsorption profile can be described by the Langmuir isotherm, which suggests monolayer adsorption. It is possible that, after sorption, large surface DNA may repel further incoming DNA molecules. Fitting the data to the Langmuir isotherm equation, X = X_∞_KC/(1 + KC), where X is the amount of DNA adsorbed per unit mass of CaPs, X_∞_ is the maximum amount of DNA that may be adsorbed, K is the Langmuir constant, C is the DNA concentration, and R^2^ is the coefficient of determination; the sorption data were linearized in C/X—C coordinates (Table 7).

Thus, CaPs obtained with cooling in the presence of 5 mM Ca^2+^ had the greatest adsorption capacity toward DNA.

### 2.3. A Release of Different Substances from CaPs

**Figure 6:**
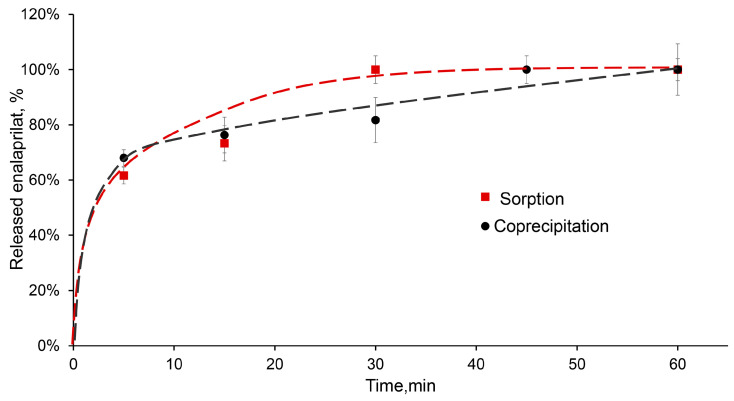
Release of enalaprilat from CaPs in saline solution, pH 7.5. Enalaprilat was included in CaPs obtained without cooling by sorption (red) and by coprecipitation (black).

The desorption of low-molecular-weight substance, enalaprilat, from CaPs obtained without cooling was studied in a saline solution at pH 7.5. Enalaprilat included in CaPs by coprecipitation almost completely released from the particles in half an hour (Figure 6). It is interesting that enalaprilat sorbed on CaPs released into the medium practically with the same kinetics, despite the different content of enalaprilat in these CaPs (Table 5) and, likely, different enalaprilat distribution throughout the particle (Figure 6).

The most suitable model for the release of enalaprilat from CaPs was the Korsmeyer–Peppas model (Table 8). It takes into account the penetration of the medium into the matrix. The constant *n* in the Korsmeyer–Peppas equation characterizes the type of diffusion and allows us to evaluate the mechanism of drug release. If the value of *n* ≤ 0.5, then the release occurs due to diffusion, obeying Fick’s laws; a value in the range of 0.5 < *n* < 1.0 indicates an anomalous transport that does not obey Fick’s laws. The release of enalaprilat from CaPs, whether coprecipitated or sorbed, was characterized by Fickian diffusion.

The release of the enzyme SOD1 coprecipitated with CaPs from the particles appeared to occur explosively; all the included SOD1 was released after 5 min of incubation in 0.15 M saline solution, which indicates low binding of the enzyme within CaPs.

**Figure 7:**
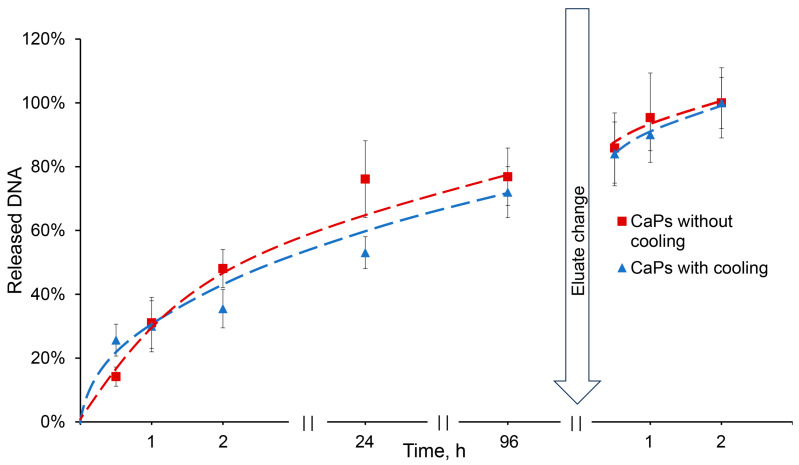
Release of DNA from CaPs obtained with and without cooling in the presence of 0.01 M citric-phosphate buffer, pH 6.5.

The release of DNA sorbed on CaPs was studied in 0.01 M citric-phosphate buffer, distilled water, and saline solution. All solutions had a pH value of 6.5. DNA release was not observed in either distilled water or saline for 24 h, but the use of citric-phosphate buffer allowed DNA to be completely washed out from CaPs (Figure 7). Apparently, this is ensured by the type of interaction between DNA and particles: the absence of the release during elution with saline indicates a non-electrostatic interaction, and the release in the presence of a phosphate anion is due to the replacement of the phosphate backbone of the DNA molecule in the complexes with PO_4_^3−^ [40].

This result is important for the putative usage of such complexes in the crop industry. There is no need to use acidic media or solutions with high salt concentrations in order to destroy the complexes; if necessary, the complexes would dissociate in the presence of a buffer, while being completely stable in water.

DNA release from CaPs obtained with/without cooling occurred in a similar manner. About 25% of the incorporated DNA was released in half an hour from CaPs obtained with cooling, while only about 15% was released from CaPs obtained without cooling. Then, the release of DNA slowed down, but after three days an equilibrium of 75% desorption was reached in both cases. After changing an elution buffer, the DNA was completely released from the particles (Figure 7).

The release data were fitted to various models (Table 9). The best-fitting model was assessed using the correlation coefficient (R^2^). As can be seen from the table, the most suitable model is the same Korsmeyer–Peppas model; other models describe the release of DNA from CaP particles as much worse. A values of *n* equal to 0.88 and 0.52 both indicate an anomalous DNA transport that does not obey Fick’s laws.

### 2.4. Studying of dsRNA Sorption on CaPs

**Figure 8:**
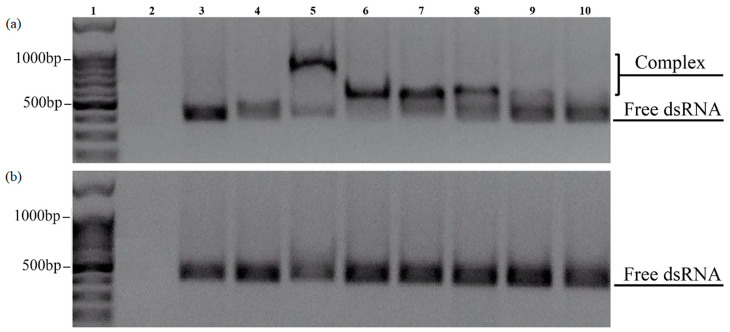
EMSA of the complexes formed between dsRNA and CaPs obtained without (**a**) and with (**b**) cooling in 1.5% non-denaturing agarose gels at various concentrations of Ca^2+^. 1—DNA ladder 100+bp; 2—CaPs; 3—dsRNA; 4—CaPs:dsRNA (5:1 *w/w*); 5—CaPs:dsRNA (10:1 *w/w*) in the presence of 10 mM CaCl_2_; 6—CaPs:dsRNA (10:1 *w/w*) in the presence of 5 mM CaCl_2_; 7—CaPs:dsRNA (10:1 *w/w*) in the presence of 1 mM CaCl_2_; 8—CaPs:dsRNA (5:1 *w/w*) in the presence of 5 mM CaCl_2_; 9—CaPs:dsRNA (2:1 *w/w*) in the presence of 5 mM CaCl_2_; 10—CaPs:dsRNA (1:1 *w/w*) in the presence of 5 mM CaCl_2_.

**Figure 9:**
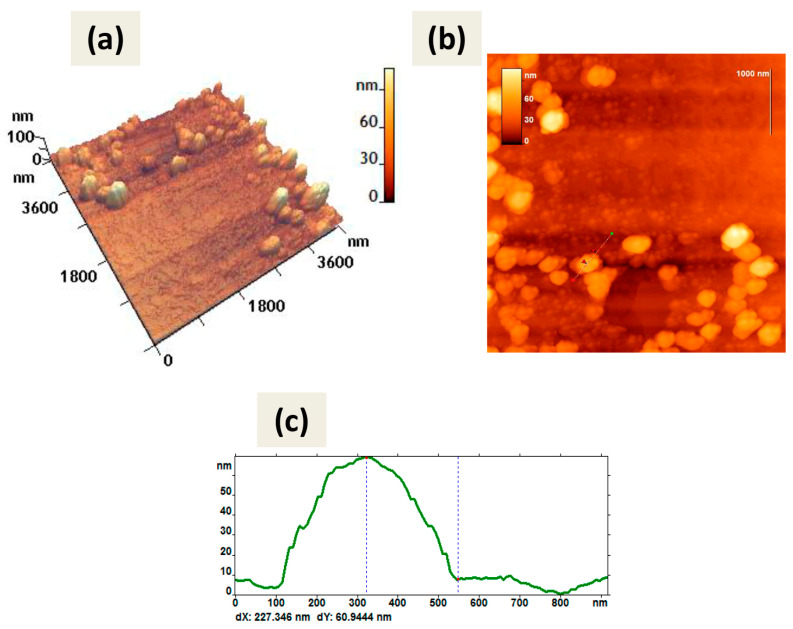
AFM images of dsRNA-CaPs complexes; CaPs were obtained without cooling. The graphite substrate was used. (**a**) 3D image, (**b**) 2D image, (**c**) cross-section of the individual particle on the 2D image. The red arrows (**b**) correspond to the dotted lines on the cross-section (**c**). The particle height according to the cross-section (**c**) is 61 nm.

The sorption of dsRNA on CaPs was carried out in the same way as for the DNA, in the presence of Ca^2+^. The obtained complexes were analyzed with electrophoretic mobility shift assay (EMSA) and studied by AFM (Figure 8 and Figure 9).

Electrophoresis showed that the CaPs pre-formed with cooling actually did not form complexes with dsRNA regardless of the presence or absence of Ca^2+^, as at different CaPs:dsRNA ratios we only observed bands corresponding to free RNA (Figure 8b), while DNA was able to bind to both types of CaPs. Thus, while the presence of a higher concentration of citrate at CaP synthesis with cooling did not affect DNA sorption to CaPs, it appeared to be critical for the sorption of RNA.

In contrast, CaPs pre-formed without cooling did form complexes with dsRNA but only in the presence of Ca^2+^ (see bands 5–8 in Figure 8a).

Moreover, an increase in the concentration of Ca^2+^ appears to significantly enhance affinity (binding) of dsRNA to preincubated CaPs, suggesting that Ca^2+^ are absolutely essential for CaPs-dsRNA binding.

The inability of CaPs obtained with cooling to bind dsRNA was confirmed by AFM, as we observed only several accumulations of dsRNA molecules and CaPs in the AFM picture on graphite substrate, whereas CaPs obtained without cooling formed complexes with dsRNA, as we observed a noticeable change in the objects’ morphology (Figure 9) compared with the original CaPs (Figure 2).

The shape of the observed objects slightly changed; they became more rounded. All geometric parameters increased significantly: perimeter (increased by one third), area (more than doubled), and roughness (almost doubled) (Table 10). The height of the observed objects also increased: the average height doubled from 35 nm to 60 nm; the maximum height increased by more than one-third. Moreover, the balls with a height of about 6–10 nm, likely belonging to dsRNA, were also visible on the surface of observed objects (Figure 9).

Thus, CaPs obtained without cooling can adsorb dsRNA and, therefore, serve as its carriers.

## 3. Materials and Methods

### 3.1. Materials

All chemicals used in this study were of analytical grade and used without further purification. Enalaprilat ((2S)-1-[(2S)-2-{[(1S)-1-carboxy-3-phenylpropyl] amino}propanoyl]pyrrolidine-2-carboxylic acid) was purchased from the U.S. Pharmacopeial Convention (Rockville, MD, USA). Recombinant human SOD1 was purchased from Life Science Advanced Technologies (St. Petersburg, Russia). DNA (sodium deoxyribonucleate, MW 270–500 kDa) from salmon milt was purchased from Immunnolex (Moscow, Russia). Angiotensin-converting enzyme (ACE) was purified from bovine lung by affinity chromatography on lisinopril-Sepharose as in [42].

The 420 bp region of Zeaxanthin epoxidase (ZEP) gene of Solanum tuberosum which is routinely used in the laboratory as a cDNA template for other purposes was selected as a random model template for synthesizing dsRNA. This fragment was cloned into the plasmid vector pAL2-T by Evrogen (Moscow, Russia). This vector contains two T7 promoters at an inverted orientation that flanks the inserted cDNA fragment. To amplify this fragment, two separate PCR reactions were conducted to get the opposite PCR products containing the T7 promoter at different ends of the original sequence. The synthesized PCR products were used as templates for in vitro RNA transcription using the Biolabmix mRNA-20 Synthesis Kit (Novosibirsk, Russia) following the manufacturer’s protocol. To form the dsRNA duplexes, equal volumes of the corresponding complementary ssRNAs were combined. The annealing buffer at the final concentration of 10 mM Tris-HCl pH 7.5, 2.5 mM MgCl_2_, 0.1 mM CaCl_2_ was added to the mixture. Then the mixture was annealed at 95 °C for 10 min and gradually cooled down to room temperature. The dsRNA was quantified using a NanoDrop spectrophotometer and examined in 1.2% agarose gels.

N-carbobenoxy-L-phenylalanyl-L-histidyl-L-leucine (Cbz-Phe-His-Leu) was purchased from Bachem AG (Bubendorf, Switzerland); o-phthalaldehyde was purchased from Sigma-Aldrich (St. Louis, MO, USA); other chemicals and reagents were from Reakhim (Moscow, Russia). Ultrapure deionized water was used for all experiments.

Before the nanoparticles synthesis, all the solutions were filtered using 0.45 µm syringe filters (Merck Millipore, Darmstadt, Germany).

### 3.2. Synthesis of Calcium Phosphate Particles

CaPs were obtained as described previously [24,27]. Briefly, a mixture of potassium phosphate and sodium citrate solutions (5:1 *v/v*) were prepared. Then, a solution of calcium chloride (5 *v*) was added simultaneously with the start of ultrasound treatment with the Bandelin Sonopuls ultrasonic homogenizer (Berlin, Germany). Initial concentrations of potassium phosphate and calcium chloride were kept constant and equal to 12.5 mM, while sodium citrate concentration, the pH value of potassium phosphate solution, and the temperature of ultrasonic exposure varied.

### 3.3. Dynamic Light Scattering (DLS) and Zeta Potential Measurements

All characteristics of CaPs, the mean hydrodynamic diameter, polydispersity index (PDI), and surface charge (ζ-potential) were measured using a Zetasizer Nano ZS setup (Malvern Co., Ltd., Malvern, UK) at 25 °C. All provided data were presented as an average of at least three values. The hydrodynamic diameter and PDI were measured via dynamic light scattering in 6 mM KCl. For ζ-potential measurements, the samples were transferred into distilled water and measured in a U-shaped cell with gold electrodes (Malvern Co., Ltd., Malvern, UK). The results were automatically processed using the Zetasizer v.7.03 software.

### 3.4. Morphology and Phase Composition of CaPs

The morphology and shape of CaPs were studied using atomic force microscopy (AFM) with a FemtoScan atomic force microscope (Moscow, Russia), and using scanning transmission electron microscopy (STEM) using scanning electron microscope Hitachi S5500 (Hitachi High-Technologies Corporation, Tokyo, Japan). Samples were pre-dialyzed against deionized water.

AFM scanning was carried out in the air in a resonant mode with a NSG10 cantilever on freshly cleaved graphite (highly oriented pyrolytic graphite) and mica substrates. We used samples of CaPs obtained with cooling (0.1 μg/μL) and without cooling (0.05 μg/μL), as well as samples of dsRNA-CaP complexes. The results were processed using FemtoScan Online software version 2.4.26 [43].

To conduct STEM, a 3 μL drop of the solution was applied to a 3 mm copper grid coated with a two-layer film of formvar and carbon; the drop was dried in air, and the grid with the sediment was kept in a vacuum at a pressure of 10^−5^ Torr for 3 h. Then the grid was moved for 12 h directly into the microscope chamber at a pressure of 10^−5^ Torr. STEM was carried out on a scanning electron microscope at an accelerating voltage of 30 kV. Electron detectors “in transmission” mode BF-STEM, and “in reflection” mode SE were used.

The phase composition was studied on a Rigaku Miniflex 600 X-ray diffractometer (Rigaku, Tokyo, Japan) in reflection mode using Cu Kα radiation and a Ni-Kβ monochromator. The suspension of CaPs was dialyzed against deionized water and lyophilized. The dried particles were re-suspended in ethanol.

### 3.5. Incorporation of Different Compounds into CaPs by Coprecipitation

Enalaprilat was loaded into CaPs at the stage of CaP synthesis (that is, by coprecipitation) with and without cooling. For this purpose, enalaprilat (7.9 mM) was dissolved in a 12.5 mM K_2_HPO_4_ solution and CaPs were obtained as described above. The weight ratio of enalaprilat to CaPs was 1.25 mg per 1 mg of particles. The percentage of enalaprilat inclusion into CaPs was determined from the difference between initial enalaprilat concentration and its concentrations in the filtrates. The suspension of enalaprilat-containing CaPs was concentrated 10 times via centrifugation on a 30 kDa membrane (Sartorius, Germany) at 5400× *g* for 5 min on a centrifuge Minispin (Eppendorf AG, Hamburg, Germany). The amount of unbound enalaprilat in the filtrates was assessed based on its ability to inhibit control of ACE activity [27]. ACE catalytic activity was measured using a fluorimetric assay with 0.2 mM of Cbz-Phe-His-Leu as a substrate in 0.15 M phosphate buffer, with pH 7.5, containing 0.15 M of NaCl and 1 μM of ZnCl_2_ at 25 °C, on the Infinite M-200 reader (Tecan, Männedorf, Switzerland). The curves of residual ACE activity on the dilution of analyzed filtrate were plotted; the dilution corresponding to 50% of ACE activity was determined and it referred to enalaprilat concentration on the preliminarily obtained calibration curve for standard enalaprilat.

SOD1 was loaded into CaPs at the stage of CaP synthesis as well. For this purpose, SOD1 (66 μg/mL or 62 kU/mL) was dissolved in 12.5 mM K_2_HPO_4_ solution and CaPs were obtained as described above. The final concentration of SOD1 in CaP suspension was 30 μg/mL. The percentage of SOD1 inclusion into CaPs was calculated by a comparison of the initial SOD1 protein and the SOD1 protein in the filtrates by the Lowry protein assay [44], as well by a comparison of initial SOD1 activity and activity in the filtrates which was determined by quercetin assay [45].

For coprecipitation of DNA and CaPs, DNA (110 μg/mL) was dissolved in a 12.5 mM K_2_HPO_4_ solution and CaPs were obtained as described above with cooling.

### 3.6. Sorption of Different Compounds on CaPs

Enalaprilat sorption on the preliminarily synthesized CaPs was performed as follows: the required amount of enalaprilat was dissolved in a suspension of CaPs with a concentration of 0.5 mg/mL. The final concentration of enalaprilat in the suspension was 3.6 mM. The amount of CaPs in the suspension was determined preliminarily, and for this purpose, an aliquot of the suspension was extensively dialyzed against distilled water, freeze-dried, and the pellet was weighed. The amount of unbound enalaprilat in the filtrates was assessed based on its ability to inhibit control of ACE activity as described above.

For SOD1 sorption on the preliminarily synthesized CaPs, SOD1 was dissolved in a CaP suspension to a final enzyme concentration of 30 or 100 μg/mL. The mixture was incubated for 2 or 4 h at room temperature, and then, for another 24 h at 4 °C. The amount of unbound SOD1 was assessed in the filtrates obtained after concentrating the suspension on 100 kDa membrane (Sartorius, Göttingen, Germany) 10 times by catalytic SOD1 activity by the quercetin method [45] and Lowry protein assay [44].

DNA sorption was carried out on CaPs in the presence of Ca^2+^ or Mg^2+^. The selection of optimal concentrations of metal cations was carried out using CaPs obtained without cooling. The concentration of CaPs was 0.93 mg/mL, and while the DNA concentration varied from 25 to 100 μg/mL, the Ca^2+^ and Mg^2+^ concentration varied in the range from 0.05 to 60 mM. The solutions of DNA and CaCl_2_ or MgSO_4_ were added to a suspension of CaPs, and sorption was carried out for 24 h at room temperature. The efficiency of DNA sorption was assessed as follows: 1 mL of CaP suspension containing DNA was centrifuged at 12,000× *g* for 10 min. The optical absorption of the supernatant was measured at a wavelength of 260 nm using Varian Cary 50 Scan UV-VIS Photometer (Varian, Palo Alto, CA, USA) and the DNA concentration was calculated using the calibration curve obtained with controlled DNA solutions.

DNA sorption isotherms were determined in both types of CaPs, obtained with and without cooling, at 5 mM of Ca^2+^ and Mg^2+^; the DNA concentration varied in a range 15–250 μg/mL, and the concentration of CaPs was 0.47 mg/mL.

To form CaPs-dsRNA complexes, dsRNA at a concentration of 50 μg/mL and a suspension of CaPs (0.47 mg/mL) obtained with and without cooling were mixed in an Eppendorf tube. The mixture was incubated for 30–60 min on ice to establish equilibrium. Then, a mixture of dsRNA and CaP suspension was applied to a 1.5% agarose gel and run at 100 V in Tris-acetate buffer for approximately 40 min. The formation of complexes of dsRNA with CaPs could be followed by the presence of the bands with lower motility in the gel.

The obtained CaPs-dsRNA complexes were analyzed with electrophoretic mobility shift assay (EMSA). To form CaPs-dsRNA complexes, dsRNA (1.125 μg per a probe) was mixed in various ratios (*w*/*w*) with suspensions of CaPs obtained with and without cooling in the presence or absence of Ca^2+^. The mixtures were incubated for 30–60 min on ice and then were subjected to electrophoresis in 1.5% non-denaturing agarose Tris-acetate gels at 100 V for 40 min. RNAs in gels were detected by staining them with ethidium bromide. The formation of CaPs-dsRNA complexes was assessed by the position (motility) of the RNA band in the gel.

### 3.7. In Vitro Release of Compounds from CaPs

Desorption of enalaprilat from CaPs was studied in 0.15 M saline solution, pH 7.5. For this purpose, several portions of a suspension of CaPs containing enalaprilat of 500 μL were concentrated 10 times via centrifugation on a 30 kDa membrane at 5400× *g* for 5 min on a Minispin centrifuge to remove 90% of the unbound enalaprilat. After that, 450 μL of 0.15 M NaCl solution, pH 7.5, was added to each concentrate, and the resulting suspension was incubated for various periods of time at room temperature. Then, the suspensions were again centrifuged and the amount of released enalaprilat was determined in each filtrate. All experiments were performed in triplicates. Mathematical analysis of experimental kinetic curves in vitro was carried out using well-known mathematical models (first order, Higuchi, Hickson–Crowell, Korsmeyer–Peppas) that describe the release of drugs from matrices of various chemical nature.

Desorption of SOD1 from CaPs was studied in 0.15 M saline solution, pH 7.5. For this purpose, several portions of a suspension of CaPs containing SOD1 of 500 μL were concentrated 10 times via centrifugation on a 100 kDa membrane at 5400× *g* for 5 min on a Minispin centrifuge to remove 90% of the unbound SOD1. After that, 450 μL of 0.15 M NaCl solution, pH 7.5, was added to each concentrate, and the resulting suspension was incubated for various periods at room temperature. Then, the suspensions were again centrifuged and the amount of released SOD1 was determined in each filtrate.

The desorption of DNA from CaPs was studied with DNA-CaP complexes obtained in an excess of Ca^2+^. The desorption of DNA was studied in 0.01 M citrate-phosphate buffer, distilled water, and saline solution with pH 6.5. For this purpose, 1 mL aliquots of CaP suspensions containing DNA were centrifuged at 12,000× *g* for 10 min on a Minispin centrifuge; the supernatant was discarded, and then 1 mL of buffer was added to the sediment and incubated for 0.5–96 h. After this time, the suspensions were again centrifuged at 12,000× *g* for 10 min, and the content of desorbed DNA in the supernatants was determined. All experiments were performed in triplicates. The mathematical analysis of experimental kinetic curves in vitro was carried out using well-known mathematical models (first order, Higuchi, Hickson–Crowell, Korsmeyer–Peppas) that describe the release of drugs from matrices of various chemical nature.

### 3.8. Statistical Analysis

Data were analyzed using Statistica for Windows (version 10.0, Stat.Soft. Inc., Tulsa, OK, USA). All data are presented as means ± SD.

## 4. Conclusions

Herein, we compared calcium phosphate nanoparticles (CaPs) obtained by two methods, with and without cooling with ice under constant ultrasound treatment. The method with cooling demanded higher concentrations of a stabilizing agent, sodium citrate, and a lower pH value of the synthesis. It was shown that both types of CaPs exist in the form of stable suspensions in water solutions, and they possess similar phase composition, with a mean hydrodynamic diameter of about 80 nm and ζ-potential −(23–25) mV. However, the synthesis of CaPs at a lower temperature resulted in obtaining a lower amount of CaPs with broader size distribution; additionally, STEM analysis showed that these CaPs existed as agglomerates of smaller nanoparticles of 10–20 nm in size.

We have shown that CaPs obtained by both methods were able to effectively incorporate a negative-charged low-molecular-weight substance, tripeptide analogue enalaprilat, at the stage of CaP formation, that is, in situ (coprecipitation). Enalaprilat inclusion into the particles is likely provided by electrostatic interaction with calcium ions inside CaPs. Enalaprilat was also able to adsorb to the preliminarily obtained CaPs, but only to those synthesized without cooling, while sorption of enalaprilat to CaPs obtained with cooling was apparently impossible. We consider this difference as an effect of higher citrate content in a later case which could impede the interactions of enalaprilat with CaPs.

Superoxide dismutase 1 (SOD1), which is also negatively charged, did not adsorb to both types of CaPs, and neither could it be incorporated into CaPs without cooling due to the thermodenaturation of the enzyme at high temperatures during ultrasonic treatment. However, SOD1 can be loaded to CaPs by coprecipitation with cooling, forming electrostatic coordination with calcium ions.

It was impossible to incorporate DNA molecules to CaPs by coprecipitation, likely due to the harmful action of ultrasound on DNA and the destruction of forming CaPs by large DNA molecules. However, DNA effectively adsorbed to the preliminarily formed CaPs, either with or without cooling, especially in the presence of Ca^2+^. RNA was also shown to adsorb to CaPs, but only to those obtained without cooling, which required less citrate during synthesis.

Thus, depending on the nature of the active agent, there are different approaches to obtaining particular agent-loaded CaPs.

The suspensions of loaded CaPs were characterized by a gradual release of the substances which make them suitable for further applications as carriers of different active agents for their use in medicine and the crop industry.

## Figures and Tables

**Table 1:** Characteristics of CaPs (mean hydrodynamic diameter and ζ-potential) obtained at different initial concentrations of sodium citrate.

C (Citrate), mM	D_h_, nm	ζ-Potential, mV
0	>1000 + precipitation	-
3.1	>1000 + precipitation	-
7.8	210 ± 20	−25 ± 2
15.6	80 ± 20	−25 ± 3
31.2	90 ± 20	−25 ± 3
46.8	100 ± 20	−24 ± 2
78.0	120 ± 10	−25 ± 3

**Table 2:** The influence of the pH and the temperature on the synthesis of CaP characteristics.

Conditions	Parameters	t, °C	Without Cooling	With Cooling
		Initial	23	4
		Final	60–70	20–23
pH 7.0	D_h_, nm		350 ± 35	>1000
	PDI *		0.51	0.63
pH 7.5	D_h_, nm		230 ± 20	>1000
	PDI		0.49	0.72
pH 8.8	D_h_, nm		80 ± 20	>1000
	PDI		0.19	-

* PDI—polydispersity index.

**Table 3:** Effect of the concentration of the stabilizing agent sodium citrate on the characteristics of CaPs obtained with ice cooling.

C (Citrate), mM		15.6	31.2	46.8
pH 7.0	D_h_, nm	>1000	530 ± 130	80 ± 20
	PDI *	0.63	0.67	0.36
	ζ, mV	-	−25 ± 2	−23 ± 4
pH 7.5	D_h_, nm	>1000	700 ± 160	160 ± 30
	PDI	0.72	0.38	0.35
	ζ, mV	-	−24 ± 2	−25 ± 3

* PDI—polydispersity index.

**Table 4:** Parameters of CaPs determined by AFM.

Sample *	Without Cooling	With Cooling
P, nm	910 ± 120	1210 ± 120
S × 10^3^, nm^2^	50 ± 13	90 ± 20
RMS, nm	7 ± 1	11 ± 1
FF 1	0.06 ± 0.02	0.09 ± 0.02
FF 2	0.8 ± 0.2	0.63 ± 0.3
H, nm	35 ± 7	44 ± 7

* The measurements were carried out on a sample of more than 10 particles separately located on the substrate. P—perimeter, length of an object’s border in the XY plane; S—area occupied by an object in the projection onto the XY plane; RMS—root mean square value of an object height (roughness); H—maximal height of an object. Form factor 1 (FF1)—the ratio of the radius of a circle of equivalent area to the radius of a circle of equivalent perimeter. For a round object, this form factor is equal to one. The more jagged the perimeter of an object, the closer its value is to zero. Form factor 2 (FF2)—the ratio of twice the length of an object’s skeleton to its perimeter. For a thin thread this ratio is equal to one, for a circle it is equal to zero.

**Table 5:** Efficiency of enalaprilat inclusion in CaPs obtained with/without cooling by coprecipitation and sorption.

Parameters	CaPs without Cooling		CaPs with Cooling	
	Sorption	Coprecipitation	Sorption	Coprecipitation
Loading, %	7.6 ± 2.2	40 ± 10	-	27 ± 4
Weight load enalaprilat/CaPs, μg/mg	160 ± 20	340 ± 30	-	250 ± 10

**Table 6:** The efficiency of DNA sorption on CaPs at different DNA concentrations in the presence of additional ions. DNA (25–100 μg/mL) sorption was carried out on CaPs (0.93 mg/mL) in the presence of 0,05 and 1 mM of Ca^2+^ or Mg^2+^. The solutions of DNA and CaCl_2_ or MgSO_4_ were added to a suspension of CaPs, and sorption was carried out for 24 h at room temperature. The efficiency of DNA sorption was calculated as the difference between the amount of DNA added and that remaining in the supernatant.

DNA Concentration, μg/mL		25	50	75	100
Mg^2+^	0.05 mM	20.3%	19.3%	11.7%	3.8%
	1 mM	34.7%	27.0%	21.3%	18.7%
Ca^2+^	0.05 mM	31.0%	18.1%	18.0%	16.8%
	1 mM	40.2%	35.3%	29.5%	25.9%

**Table 7:** Calculation of thermodynamic parameters of DNA sorption on CaPs.

Parameter	CaPs Without Cooling in the Presence of 5 mM Mg^2+^	CaPs Without Cooling in the Presence of 5 mM Ca^2+^	CaPs with Cooling in the Presence of 5 mM Ca^2+^
X_∞,_ μg	98.0	131.6	149.3
K, μg^−1^	7.8 × 10^−3^	6.1 × 10^−3^	5.9 × 10^−3^
R^2^	0.99	0.95	0.99

**Table 8:** Data approximation using various mathematical models for the release of enalaprilat from CaPs.

Model	Equation *	Parameter	Coprecipitation	Sorption
Korsmeyer–Peppas	$\frac{M_{t}}{M_{\infty }}=K_{KP}\cdot t^{n}$	K	0.58	0.39
		*n*	0.10	0.26
		R^2^	0.99	0.92
Higuchi	$\frac{M_{t}}{M_{\infty }}=K_{H}\cdot t^{\frac{1}{2}}$	K	4.8 × 10^−2^	7.6 × 10^−2^
		R^2^	0.86	0.84
First order	$\ln\left(1-\frac{M_{t}}{M_{\infty }}\right)=K_{1}\cdot t$	K	9.1 × 10^−3^	8.6 × 10^−3^
		R^2^	0.96	0.73
Hixson–Crowell	${\left(1-\frac{M_{t}}{M_{\infty }}\right)}^{\frac{1}{3}}=1-K_{\beta }\cdot t$	K	2.5 × 10^−3^	3.5 × 10^−3^
		R^2^	0.95	0.87

* Where *M_t_/M_∞_* is a fraction of drug released at time *t*; *K_KP_* is the Korsmeyer–Peppas release rate constant; *n* is the release exponent; *K_H_* is the Higuchi release rate constant; *K*_1_ is the first-order release rate constant; *K_β_* is the Hixson–Crowell release rate constant.

**Table 9:** Data approximation using various mathematical models for the release of DNA adsorbed on CaPs.

Model	Equation *	Parameter	Coprecipitation	Sorption
Korsmeyer-Peppas	$\frac{M_{t}}{M_{\infty }}=K_{KP}\cdot t^{n}$	K	2.8 × 10^−3^	3 × 10^−3^
		*n*	0.88	0.52
		R^2^	0.97	0.99
Higuchi	$\frac{M_{t}}{M_{\infty }}=K_{H}\cdot t^{\frac{1}{2}}$	K	1.2 × 10^−3^	6 × 10^−4^
		R^2^	0.85	0.96
First order	$\ln\left(1-\frac{M_{t}}{M_{\infty }}\right)=K_{1}\cdot t$	K	4.7 × 10^−2^	2.5 × 10^−2^
		R^2^	0.55	0.86
Hixson-Crowell	${\left(1-\frac{M_{t}}{M_{\infty }}\right)}^{\frac{1}{3}}=1-K_{\beta }\cdot t$	K	6.8 × 10^−5^	3.7 × 10^−5^
		R^2^	0.77	0.92

* Where *M_t_/M_∞_* is a fraction of drug released at time *t*; *K_KP_* is the Korsmeyer–Peppas release rate constant; *n* is the release exponent; *K_H_* is the Higuchi release rate constant; *K*_1_ is the first-order release rate constant; *K_β_* is the Hixson–Crowell release rate constant.

**Table 10:** Morphology characteristics of CaPs-dsRNA complexes in comparison with CaPs.

Sample	CaPs	Complex CaPs-dsRNA
P, nm	910 ± 120	1330 ± 160
S × 10^3^, nm^2^	50 ± 13	100 ± 30
RMS, nm	7 ± 1	13 ± 4
FF 1	0.06 ± 0.02	0.09 ± 0.004
FF 2	0.8 ± 0.2	0.65 ± 0.15
H, nm	35 ± 7	60 ± 15

## Data Availability

The data supporting the findings of this study are available within the article.
